# Comparing Psychiatric Admission Following Suicidal Presentations to the Emergency Department pre-COVID and During the COVID era

**DOI:** 10.1192/j.eurpsy.2024.555

**Published:** 2024-08-27

**Authors:** S. Crowley, E. Cassidy

**Affiliations:** ^1^Clare Psychiatry of Later Life, HSE Mid West, Ennis; ^2^Department of Liaison Psychiatry, Cork University Hospital, Cork, Ireland

## Abstract

**Introduction:**

Suicide is one of the leading causes of death worldwide, (Centiti et al. 2020). Presentations to the emergency department (ED) with suicidal ideation (SI) or deliberate self-harm (DSH), and admissions following same, are a major part of unscheduled adult mental health service activity.

**Objectives:**

To evaluate how suicidal presentations to the emergency department (ED), and admission following same have been affected by the COVID era thus far. To evaluate how key patient characteristics affect admission during the COVID era and pre-COVID, namely whether presentations were with suicidal ideation (SI) or deliberate self-harm (DSH), whether the patient was previously known to a community mental health team (CMHT), and whether the patient was intoxicated at the time of presentation.

**Methods:**

Data is routinely collected on all adults presenting with SI/DSH to the ED. We looked at presentations, admissions and key patient characteristics over the 12 months of the COVID era thus far (March 2020-February 2021) and compared them to the preceding 12 months.

**Results:**

Presentations over the two 12 month periods were similar (pre-COVID n=819, COVID era n=823). However, admission increased by 27% (139 to 177) over the COVID era as a whole. For nine months of the COVID era monthly numbers of admissions were higher than their pre COVID comparison. Admission rates during the COVID era were found to be increased across all patient groups examined, but were particularly increased in those presenting sober or with SI. Admission rates rose equivalently for those known or unknown to a CMHT.

**Image:**

**Figure:**
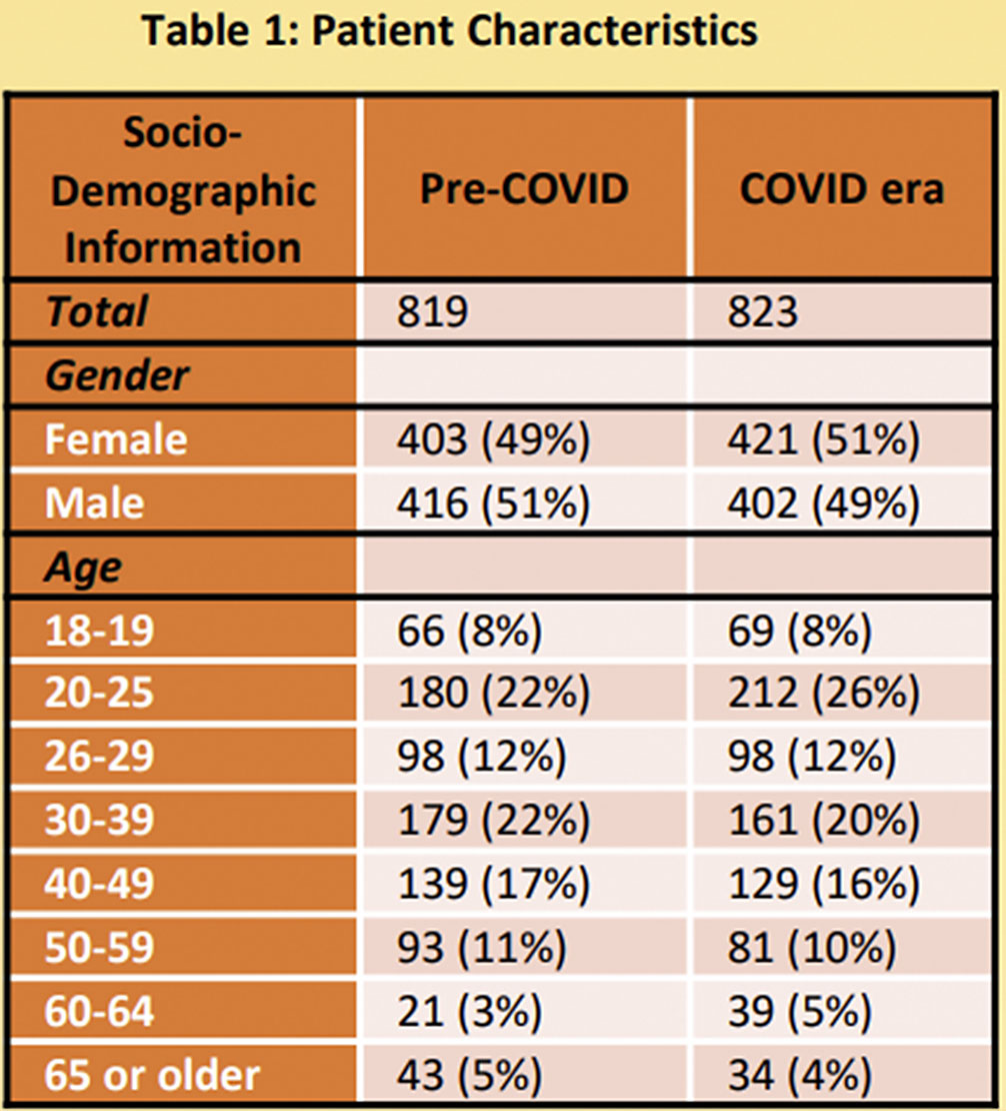

**Image 2:**

**Figure:**
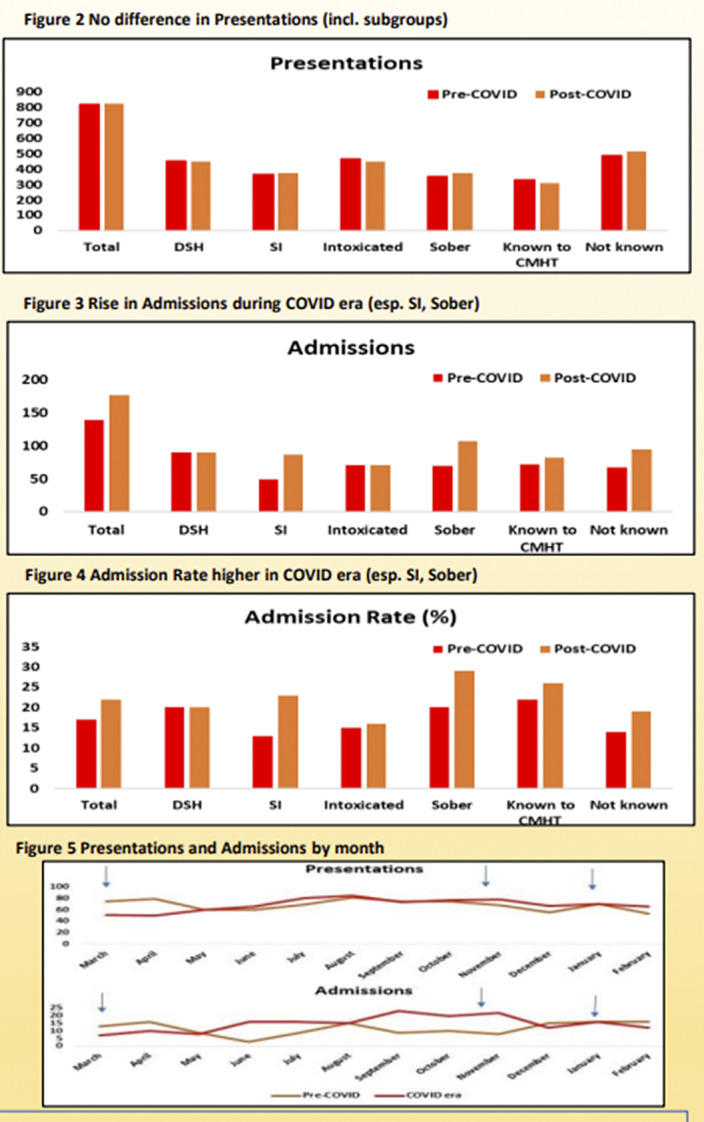

**Conclusions:**

The number of admissions following suicidal presentations to the ED has risen significantly in the COVID era. This may be due to more severe presentations in terms of risk of suicide without admission or increased psychiatric morbidity requiring admission. Limitations of service provision in the community due to COVID era restrictions may also partially explain these findings.

**Disclosure of Interest:**

None Declared

